# Prodigiosin Promotes Nrf2 Activation to Inhibit Oxidative Stress Induced by Microcystin-LR in HepG2 Cells

**DOI:** 10.3390/toxins11070403

**Published:** 2019-07-12

**Authors:** Jihua Chen, Yuji Li, Fuqiang Liu, De-Xing Hou, Jingjing Xu, Xinying Zhao, Fei Yang, Xiangling Feng

**Affiliations:** 1 Xiangya School of Public Health, Central South University, Changsha 410128, Hunan, China; 2 Department of Public Health Emergency Treatment, Hunan Center for Disease Control and Prevention (CDC), Changsha 410005, Hunan, China; 3 Department of Food Science and Biotechnology, Faculty of Agriculture, Kagoshima University, Kagoshima 890-0065, Japan

**Keywords:** prodigiosin, microcystin-LR, ROS, Nrf2, ubiquitination

## Abstract

Microcystin-LR (MC-LR), a cyanotoxin produced by cyanobacteria, induces oxidative stress in various types of cells. Prodigiosin, a red linear tripyrrole pigment, has been recently reported to have antimicrobial, antioxidative, and anticancer properties. How prodigiosin reacts to reactive oxygen species (ROS) induced by MC-LR is still undetermined. This study aimed to examine the effect of prodigiosin against oxidative stress induced by MC-LR in HepG2 cells. Ros was generated after cells were treated with MC-LR and was significantly inhibited with treatment of prodigiosin. In prodigiosin-treated cells, the levels of nuclear factor erythroid 2-related factor 2 (Nrf2) and Nrf2-related phase II enzyme heme oxygenase-1 (HO-1) were increased. Besides, prodigiosin contributed to enhance nuclear Nrf2 level and repressed ubiquitination. Furthermore, prodigiosin promoted Nrf2 protein level and inhibited ROS in Nrf2 knocked down HepG2 cells. Results indicated that prodigiosin reduced ROS induced by MC-LR by enhancing Nrf2 translocation into the nucleus in HepG2 cells. The finding presents new clues for the potential clinical applications of prodigiosin for inhibiting MC-LR-induced oxidative injury in the future.

## 1. Introduction

Cyanobacteria (blue green algae) are photosynthetic bacteria that breed in fresh, braskish, and marine waters [1], causing health hazards to fish, shellfish, crops, and even people given certain conditions. Massive blooms of cyanobacterial are considered as a worldwide environmental problem [2]. During the algae blooms, cyanobacteria produce cyanotoxins, which may contaminate drinking water and lead to adverse health effects [3]. Microcystins (MCs) are the most prevalent cyanotoxin according to reports. They have been proven to be a threat to aquaculture [4] and agriculture [5], polluting lakes and reservoirs [6]. Surveys conducted in United States and Canada showed that 80% of source and treated water samples were contaminated by MCs [7]. Similar results were generalized from surveys in Europe, reporting that MCs comprised 60% of detected cyanotoxins in fresh water [8]. Among MCs, microcystin-LR is most commonly detected in Europe, America, and East Asia. MC-LR is known to induce DNA damage [9] and up-regulate important signaling pathways inducing hepatotoxicity [10,11]. Some studies demonstrated that MC-LR could be transported into liver cells by organic anion polypetide transporters (OATPs) and inhibited protein phosphatase 1 and 2A (PP1 and PP2A) [12,13,14,15]. Besides, MC-LR was reported to induce reactive oxygen species (ROS) production, then destroying the cytoskeleton and resulting in cell necrosis and apoptosis [16,17].

Prodigiosin is a red bacterial pigment that is secreted as a secondary metabolite of *Serratia marcescens*. It is reported as the most prominent member of the prodiginine family (including undecylprodigiosin, cycloprodigiosin, metacycloprodigiosin, and prodigiosin R1) [18]. Previous studies demonstrated that *Serratia marcescens* could inhibit the growth of *Anabaena* [19] and *Microcystis aeruginosa* [20] by producing prodigiosin. Due to its many potential benefit properties, including anticancer [21], antimicrobial [22], and antimalarial [23] effects, prodigiosin has been a subject of interest. Besides those bioactivities mentioned above, recent studies have begun to uncover the antioxidative activity of prodigiosin. It was reported that prodigiosin could effectively scavenge the free radicals in food stuffs [24]. However, the mechanism of prodigiosin for inhibiting ROS has not been reported in detail.

Nuclear factor erythroid 2-related factor 2 (Nrf2) is a transcription factor active in response to oxidative stress. It significantly enhances the antioxidative defense of stressed cells and contributes to the elimination of exogenous chemicals and their toxic metabolite through elevating expression of down-stream phase II detoxification enzymes [25]. Under the consideration of severe oxidative injury induced by MC-LR, Nrf2 might be a crucial factor to control MC-LR caused intracellular damage. In this study, to investigate whether prodigiosin could inhibit oxidative stress induced by MC-LR through enhancing Nrf2, we treated HepG2 cells with MC-LR and prodigiosin and determined the ROS level, intensity of Nrf2-related protein, and translocation of Nrf2. Furthermore, Nrf2 knocked-down HepG2 cells were used to investigate the effect of MC-LR and prodigiosin on HepG2 with low level of Nrf2. The results of the study could provide new clues for the mechanism of how prodigiosin inhibits oxidative stress caused by MC-LR.

## 2. Results

### 2.1. Effect of ROS Level Induced by MC-LR and Prodigiosin

**Figure 1:**
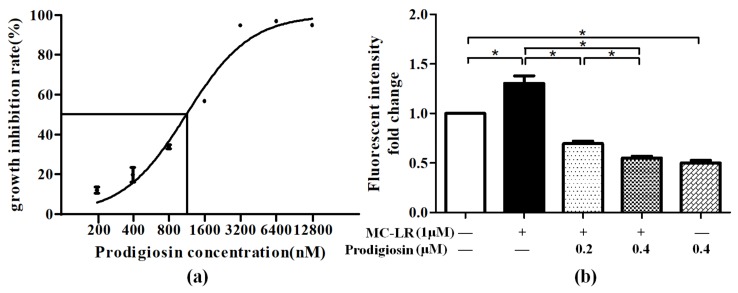
(**a**) Cell viability of HepG2 after prodigiosin (200, 400, 800, 1600, 3200, 6400, 12,800 nM) treatment for 24 h. (**b**) Effects of microcystin-LR (MC-LR) (1 μM) and prodigiosin (0.2 and 0.4 μM) on intracellular reactive oxygen species (ROS) production. Values are presented as mean ± SD. * *p* < 0.05.

HepG2 cells were exposed to different concentrations of prodigiosin for 24 h and a cell counting kit-8 (CCK8) assay was performed. Calculated by graphpad prism 5, the IC_50_ of HepG2 treated with prodigiosin was 1124 nM. After cells were treated with 0.2 μM and 0.4 μM prodigiosin, proliferation in nearly 12.1% and 19.9% of cells, respectively, was inhibited. With the increasing concentration of prodigiosin, the cell growth inhibition rate rose accordingly. When the concentration exceeded 3.2 μM, the growth inhibition rate reached a plateau of about 95% (Figure 1a).

Intracellular reactive oxygen species content was determined by a fluorogenic probe, 2′, 7′-dichlorofluorescin diacetate (DCFH-DA). Compared with control group, it was observed that fluorescent intensity was significantly increased by 1.3-fold after cells were treated with MC-LR and declined by nearly 2-fold in the cells treated with prodigiosin in dose-dependent manner (Figure 1b).

### 2.2. Effect of Prodigiosin and MC-LR in Nrf2-Related Protein

**Figure 2:**
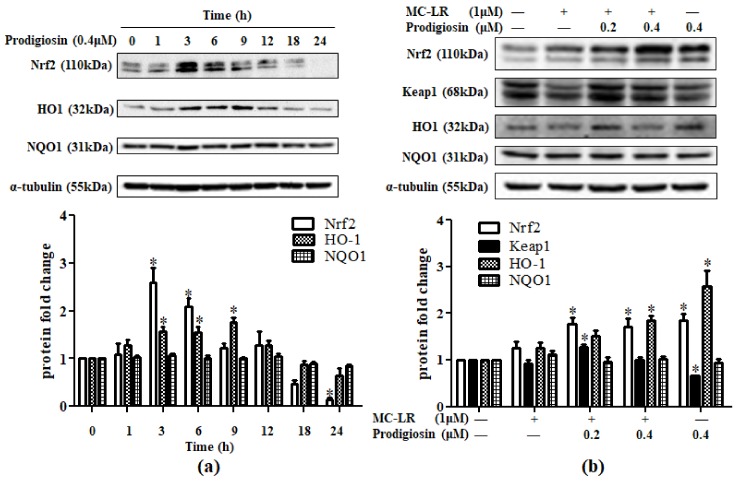
(**a**) Prodigiosin (0.4 μM) affected the level of nuclear factor erythroid 2-related factor 2 (Nrf2) and phase II enzyme in time dependence. (**b**) Effects of MC-LR (1 μM) and prodigiosin (0.2 and 0.4 μM) on the levels of Nrf2, Keap1, heme oxygenase-1 (HO-1), and NADP(H): ubiquinone oxidoreductase (NQO1) protein. Values were presented as mean ± SD. * *p* < 0.05 vs. the relative control group.

The level of protein in the Nrf2/Keap1 pathway was determined by Western blot. After treatment with prodigiosin, level of Nrf2 and heme oxygenase-1 (HO-1) was significantly increased at the highest level at 3 h for Nrf2 and 9 h for HO-1 and declined afterward, while NADP(H): ubiquinone oxidoreductase (NQO1) levels were comparatively unchanged (Figure 2a). Besides, protein levels of Nrf2 and HO-1 were increased in a prodigiosin dose-dependent manner. Keap1 was expressed highly in the low-dose prodigiosin group but decreased with rising doses. NQO1 showed no significant change (Figure 2b).

### 2.3. Prodigiosin and MC-LR Affect the Translocation of Nrf2

#### 2.3.1. Activation of Nrf2 in the Nucleus

**Figure 3:**
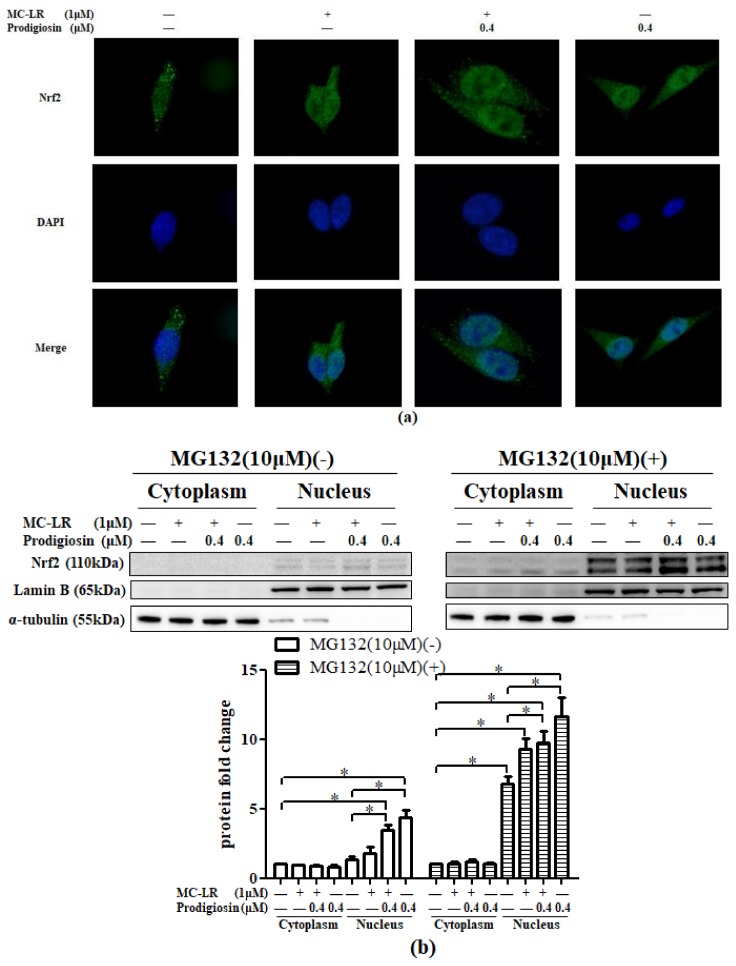
(**a**) Effects of prodigiosin (0.4 μM) and MC-LR (1 μM) on Nrf2 translocation into the nucleus performed with immunofluorescence analysis; (**b**) Effects of prodigiosin (0.4 μM) and MC-LR (1 μM) on nuclear Nrf2 accumulation performed with Western blot analysis. Values are presented as mean ± SD. * *p* < 0.05 vs relative control group.

To investigate whether prodigiosin or MC-LR could affect Nrf2 translocation, immunofluorescence assay, and Western blot were performed to detect Nrf2 level in the plasm and nucleus of HepG2 cells. Through immunofluorescence staining, the Nrf2 (green)-stained fluor-conjugated secondary antibody (Figure 3a, upper image), the nucleus (blue) stained with 4’,6-diamidino-2-phenylindole (DAPI) (Figure 3a, middle image), and the merged image of MC-LR and prodigiosin-treated cells (Figure 3a, lower image) showed the nuclear location of Nrf2 protein. MC-LR slightly increased the intranuclear level of Nrf2, and prodigiosin exerted this effect better. This was confirmed by Western blot analysis (Figure 3b left), showing remarkably higher levels of Nrf2 in the nucleus of the prodigiosin-treated group as compared to the cells treated in the control group. MG132 was pretreated to inhibit the degradation of Nrf2 so the nuclear translocation would be more obvious (Figure 3b right). The result was similar to that of the non-MG132-treated group.

#### 2.3.2. Inhibition of Ubiquitination by Prodigiosin and MC-LR

**Figure 4:**
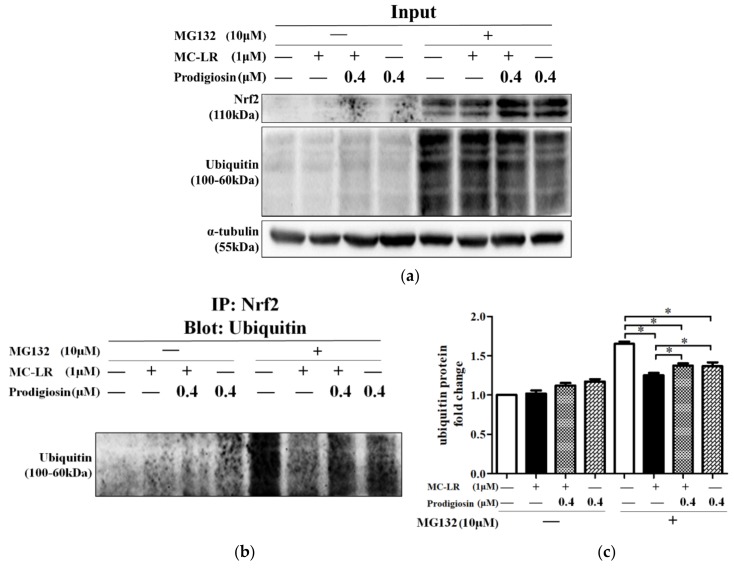
Effects of MG132 (10 μM), prodigiosin (0.4 μM), and MC-LR (1 μM) on Nrf2 ubiquitination. (**a**) Protein extracted normally was detected by Western blot, and was used as a control. (**b**) Nrf2 protein was extracted after immunoprecipitation with Nrf2 and was then detected by Western blot with the ubiquitin antibody. (**c**) Quantification of ubiquitin protein bands. Values are presented as mean ± SD. * *p* < 0.05 vs. relative control group.

Unactivated Nrf2 is degraded through the ubiquitin-26S proteasome pathway in cytoplasm. We next investigated the mechanism of Nrf2 by examining the ubiquitination of Nrf2 in HepG2 cells. Total protein was normally extracted as input group (Figure 4a). Nrf2 and its conjoint ubiquitin were extracted by immunoprecipitation (Figure 4b). Pretreated with MG132, Nrf2 ubiquitination was greatly preserved (fifth column in Figure 4c). This effect was significantly declined with the treatment of MC-LR and prodigiosin (sixth to eighth columns in Figure 4c), indicating that MC-LR and prodigiosin might strongly affect the binding between ubiquitin and Nrf2.

### 2.4. Effect of Prodigiosin and MC-LR on Nrf2 Knocked Down Cells.

**Figure 5:**
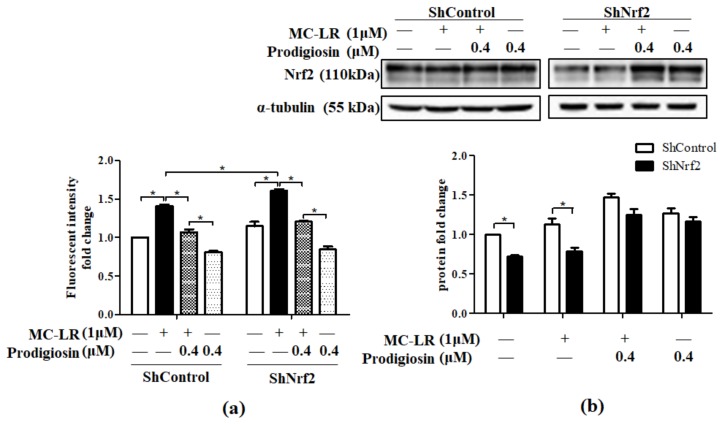
(**a**) Effects of MC-LR (1 μM) and prodigiosin (0.4 μM) on intracellular ROS production in Nrf2-knocked down HepG2 cells. Data were determined by adopting spectrofluorimetry. (**b**) Effects of MC-LR (1 μM) and prodigiosin (0.4 μM) on Nrf2 level in Nrf2-knocked down HepG2 cells. Results were shown as Western blot for control ShRNA and Nrf2 ShRNA. Values were presented as mean ± SD. * *p* < 0.05.

To discover the effect of prodigiosin and MC-LR on Nrf2, we further transfected HepG2 cells with Nrf2 knocked-down short hairpin ribonucleic acid (ShRNA) plasmid. Compared with control group, results of ROS assay indicated MC-LR led to high fluorescent intensity by 1.5 fold, while prodigiosin significantly attenuated fluorescent intensity by about 1.3-fold (Figure 5a). Western blot further demonstrated that MC-LR could not change the level of Nrf2 significantly, while prodigiosin elevated Nrf2 level back to the normal level (Figure 5b).

## 3. Discussion

Oxidative stress has been implied to be a crucial factor in numerous diseases, such as cancer, ischemia, and neurodegenerative disorders [26]. The increasing level of ROS in cells can be a signal for cell death or the autophagy pathway [27]. Among the toxicants that lead to serious oxidative injury, cyanotoxin is considered to be one of the most dangerous and broadly affected. As the most prevalent cyanotoxin in the environment, MC-LR has been widely studied for its induction of oxidative stress in various organs of different animal species. For instance, a study of female zebrafish showed that MC-LR could induce the overproduction of ROS and eventually lead to apoptosis [28]. In order to suppress such effects, new detoxicants are studied to identify the compounds and mechanism against ROS, such as quercetin [29] and naringin [30]. In this study, prodigiosin, a red bacterial pigment, was used as an antagonist against ROS. The biological activities it has, including anticancer, immunosuppression, and antimalarial effects, contribute to wide range of studies. How prodigiosin responses to ROS in animal cells remains to be seen. Consequently, a hepatic carcinoma cell line (HepG2) was treated with MC-LR to induce intracellular ROS, followed by prodigiosin treatment to identify the antioxidative effect of this pigment on animal cells. After the IC_50_ of prodigiosin was ascertained as 1124 nM, we chose 0.2 and 0.4 μM as the experimental concentrations of prodigiosin. A result screened from the National Cancer Institute Drug Discovery Program demonstrated that prodigiosin has an average IC_50_ of 2.1μM in 60 kinds of human tumor cells, which was much higher than the concentration we chose. Besides, the concentration of MC-LR was selected as 1 μM according to previous reports [31]. Under such concentrations, prodigiosin and MC-LR treatment apparently did not affect the growth of HepG2 cells. In the ROS assay, ROS levels of HepG2 cells treated with prodigiosin were significantly lower than in cells only treated with MC-LR, which indicated that prodigiosin might attenuate oxidative injury caused by MC-LR. Similar outcome was reached in an experiment exerted on mice, showing that prodigiosin inhibited oxidative injury induced by hypoxia [32]. To identify how prodigiosin inhibits ROS induced by MC-LR in HepG2 cells, Nrf2, an essential transcription factor of regulating the expression of antioxidative genes in response to oxidative stress, needs to be clarified. 

The Keap1/Nrf2 signaling pathway is a crucial mechanism to protect cells from oxidative stress [33]. In basic conditions, Keap1 binds Nrf2 in the cytoplasm, facilitating the ubiquitination and degradation of Nrf2 in 26S proteasome to maintain Nrf2 at low level [34]. When the signaling pathway is activated by oxidative stress, inflammation, or injury, Nrf2 is released from Keap1 and transfers into nucleus, where it combines with small Maf proteins and binds to the antioxidative responsive elements, consequently initiating downstream phase II detoxification enzymes, including heme-oxygenase-1 (HO-1), NADP(H): ubiquinone oxidoreductase (NQO1), glutathione-S-transferase (GST) [35]. Phase II enzymes play an important role in antioxidation. For instance, HO-1 shows cytoprotective activity through converting free heme to biliverdin, Fe^2+^ and CO. Subsequently, biliverdin converts to bilirubin, Fe^2+^ increases level of antioxidative ferritin, and CO produces anti-inflammatory and anti-apoptotic effect. In this study, the protein level of Nrf2 did not change significantly after treated with MC-LR for 1 h. This might indicate that the rising ROS level induced by MC-LR was not related to Nrf2. The results in the 6-h prodigiosin-treated group indicated that increasing level of HO-1 might account for ROS declination, in accordance with a previous review which reported that Nrf2 and HO-1 play an important role in responsible for oxidative injury [36]. Interestingly, we found that Keap1, the Nrf2 regulatory protein, was down regulated with prodigiosin treatment (Figure 2b). Since Keap1 restricts the translocation of Nrf2, decreasing level of Keap1 could herald the activation of Nrf2 in the nucleus.

Previous study demonstrated that overexpression of Nrf2 and its target gene might be considered as biomarkers of cells injury [37]. According to the upregulated level of Nrf2 and hypothesis of Nrf2 activation mentioned above, the level of Nrf2 in the cytoplasm and nucleus needs to be explored. As immunofluorescence image development showed, prodigiosin contributed to the uptrend of Nrf2 located in the nucleus. This was further confirmed by Western blot, showing high nuclear level of Nrf2 in cells treated with prodigiosin. Observed by former researchers, MC-LR could induce Nrf2 transportation into the nucleus. A possible explanation might be that MC-LR binds to Keap1 through competition with Nrf2 and then liberates Nrf2 [38]. But in this study, MC-LR didn’t significantly contribute to Nrf2 translocation. This might be due to different concentrations of MC-LR and type of cell we used. We found that Nrf2 protein level of cells treated with prodigiosin was the highest among the treatment groups, which might confirm that prodigiosin contributed to the translocation of Nrf2. Effective as prodigiosin might be, the combination between Nrf2 and ubiquitin (degradation biomarker of Nrf2) under prodigiosin and MC-LR interventions remained undiscovered. To confirm the interaction between ubiquitin and Nrf2, immunoprecipitation experiments were carried out and results suggested that the binding of Nrf2 and ubiquitin was enhanced with treatment of MG132, but it was weakened after being treated with MC-LR and prodigiosin (Figure 4). Unbinding between Nrf2 and ubiquitin suggested Nrf2 was released from plasm and transferred into nucleus, based on the results of cytoplasmic and nuclear protein Western blot. Therefore Nrf2 was released and triggered the overexpression of genes in the pathway. In another study on p47^phox^, a cytosolic subunit of NADPH oxidase, was similarly concluded that p47^phox^ activated Nrf2 through attenuating ubiquitination. They raised an interesting hypothesis that p47^phox^ bound to Nrf2, consequently ubiquitination mediated by Keap1 was interfered and Nrf2 began accumulating in the nucleus [39]. In addition to interpret the way Nrf2 was activated, it provided a new thought of how Nrf2 react to exogenous compound. Further study of prodigiosin is needed to investigate the interaction mechanism between prodigiosin and Nrf2-related protein.

Since Nrf2 acts a specific role in cytoprotection, whether could cells resist ROS when Nrf2 is knocked down must be considered. A study about sulforaphane-inhibited toxicity induced by MC-LR demonstrated that antioxidative activities were abolished when Nrf2 was knocked down in NIH 3 T3 cells [40]. In present study, the significant difference of ROS level observed in ShControl and ShNrf2 cells treated with MC-LR indicated the absence of Nrf2 might lead to higher ROS levels when the cells were affected by MC-LR. After treated with prodigiosin, ROS level declined compared to MC-LR treated cells but did not differ significantly among prodigiosin-treated ShControl and ShNrf2 groups, suggesting prodigiosin inhibited ROS induced by MC-LR and might promote Nrf2 to inhibit ROS in ShNrf2 cells (Figure 5a). Results of Western blot confirmed that prodigiosin treatment promoted the expression of Nrf2 back to normal level, while MC-LR could not significantly bring any effect (Figure 5b), indicating prodigiosin could restore Nrf2 level and therefore attenuate ROS in Nrf2 knocked-down cells.

In conclusion, we have shown that MC-LR can overproduce ROS and slightly increase translocation of Nrf2 into nucleus. Acting as the antagonist against MC-LR, prodigiosin significantly boosts Nrf2 entry to the nucleus by inhibiting ubiquitination degradation and consequently activates antioxidative enzymes. In Nrf2 knocked-down cells, prodigiosin is able to rebound Nrf2 and therefore might scavenge ROS. Results suggest that prodigiosin presents the protective mechanism against oxidative stress and reduces damage caused by MC-LR through activating Nrf2. 

## 4. Materials and Methods 

### 4.1. Cell Culture and Treatment

HepG2 cells were obtained from Cancer Research Institute of Central South University (Changsha, China) and cultured in RPMI 1640 medium (Gibco, Grand Island, NY, USA) supplemented with 10% FBS (Gibco, Grand Island, NY, USA) and penicillin–streptomycin solution (Gen-View, Calimesa, CA, USA) in a humidified incubator at 37 °C and 5% CO_2_. HepG2 cells were seeded in 6-cm-dish at 37 °C for 20 to 28 h; then prodigiosin (Enzo Life sciences, New York, NY, USA) stock solution was added directly to cell culture media at a final concentration of 0.2 or 0.4 μM for 6 h. In the last 1 h, MC-LR (Taiwan Algal Science Inc., Taiwan, China, purity ≥95%) stock solution was added directly to cell culture media at a final concentration of 1 μM for 1 h. 

### 4.2. CCK8 Assay

The CCK8 assay was performed to assess the impact on cell viability. Cells were seeded in 96-well plates at a concentration of 4 × 10^3^ cells/well, cultured for 16 h and subsequently treated with different concentrations of prodigiosin (0.2, 0.4, 0.8, 1.6, 3.2, 6.4, and 12.8 μM) for 24 h. Then, 10 μL CCK8 (biotool, Kirchberg, Switzerland) was added to each well, and cells were incubated for 3 h at 37 °C. Absorbance was measured using a microplate reader (BioTek, Winooski, VT, USA) at the wavelength of 450 nm. Cell viability was expressed as percentage of CCK8 reduction. Each experiment was repeated three times.

### 4.3. ROS Assay

The formation of intracellular ROS was determined using DCFH-DA (Sigma-Aldrich, St. Louis, MO, USA). HepG2 cells were seeded at a density of 3 × 10^5^ into 3.5 cm-dish. After 24 h of incubation at 37 °C in 5% CO2, RPMI 1640 medium was refreshed and cells were treated with prodigiosin (0.2, 0.4 μM) for 5 h. Then cells were treated with MC-LR (1 μM) for 1 h. After that, the culture medium was refreshed again and DCFH-DA was added. After 1 h, culture medium was removed and cells were preserved in PBS. Spectrofluorimeter (PerkinElmer, Waltham, MA, USA) was used to detect the fluorescence at excitation wavelength of 485 nm and emission wavelength at 525 nm.

### 4.4. Western Blot Analysis

The Nuclear and Cytoplasmic Protein Extraction Kit (Beyotime, Shanghai, China) was used for nuclear and cytoplasmic proteins extraction. Cell were harvested and lysed in radio immunoprecipitation assay (RIPA) buffer (Beyotime, Shanghai, China) with protease inhibitor phenylmethylsulfonyl fluoride (PMSF) (Beyotime, Shanghai, China). The lysates were centrifuged at 10,000 *g*, 4 °C, for 15 min. Protein concentration was determined by Ultra-micro spectrophotometer (Implen, Munich, Germany). Proteins were separated on 10% sodium dodecyl sulfate-polyacrylamide gel electrophoresis (SDS-PAGE) and transferred onto polyvinylidene difluoride (PVDF) membranes. Membranes were blocked with 5% non-fat milk for 1 h and incubated with primary antibodies overnight at 4 °C. Then the membranes were washed with Tris-Buffered Saline Tween20 (TBS-T) and incubated with secondary antibody for 1.5 h. Chemiluminescence imaging system (Tanon 5500, Shanghai, China) was used to detect the membranes. Protein levels were standardized by comparison with Lamin B (Santa Cruz, Dallas, CA, USA) or α-tubulin (ABclonal, Boston, MA, USA). Representative blots were chosen from three independent experiments.

### 4.5. Immunofluorescence Assay

HepG2 cells (4 × 10^4^/well) were cultured on coverslips in 24-well plates for 24 h. Then cells were treated with prodigiosin (0.4 μM) for 6 h and MC-LR (1 μM) for 1 h. After treatment, cells were fixed using 4% paraformaldehyde solution (Dingguo, Beijing, China) for 40 min, permeabilized with 0.1% Triton-X (Solarbio, Beijing, China) for 20 min, and blocked with Goat Serum (Boster, Wuhan, China) for 1 h. Then, cells were incubated with Nrf2 primary antibody (Abcam, Cambridge, UK) at 4 °C overnight. After that, cells were incubated with fluorescent secondary antibody for 2 h at room temperature and stained with DAPI for 5 min. Finally, coverslips were mounted using antifade mounting medium (Boster, Wuhan, China) on slides, observed, and photographed using fluorescence microscope (ZEISS, Jena, Germany).

### 4.6. Immunoprecipitation

After treatment with prodigiosin (0.4 μM) for 6 h and MC-LR (1 μM) for 1 h, HepG2 cells were lysed with cell lysis buffer containing 1 mM PMSF. The lysates were stirred at 4 °C for 2 h, and the homogenates were centrifuged at 12,000 *g* for 15 min at 4 °C. The protein concentrations of supernatants were determined using Ultra-micro spectrophotometer. For immunoprecipitation, the cell extracts (0.5 mg) were pre-incubated with 2 μg normal rabbit immunoglobulin G (IgG) and 20 μL Protein A agarose beads for 2 h to reduce nonspecific reactions. The mixture was centrifuged at 4 °C at 2500 rpm for 5 min. After that, the supernatants were incubated with 2 μg of anti-Nrf2 antibody (Dingguo, Beijing, China) at 4 °C overnight and then mixed with 30 μL Protein A agarose beads (Beyotime, Shanghai, China) at 4 °C for 3 h. Then, immunoprecipitation solutions were centrifuged at 2500 rpm for 5 min at 4 °C to collect the beads, and the beads were washed once with cell lysis buffer. The complexes were eluted with 30 μL SDS loading buffer, heated at 100 °C for 5 min, and analyzed by Western blotting.

### 4.7. Transfection

ShRNA (ccggacTGACAGAAGTTGACAATTActcgagTAATTGTCAACTTCTGTCAgttttt-tg) targeting Nrf2 (GeneChem, Shanghai, China) was designed and cloned into double-marked lentivirial vector GV248 (GeneChem, Shanghai, China). The plasmids were generated and transfected into HepG2 using Lipofectamine 3000 (Thermo Fisher Scientific, Shanghai, China). Non-specific cells were eliminated with puromycin treatment and sorted by flow cytometry. Transfected cells were cultured and examined with ROS assay and Western blot.

### 4.8. Statistical Analysis

The experimental results were expressed as the mean ± SD of at least three independent experiments. Statistical analyses were performed with SPSS 18.0 (Chicago, IL, USA). Data were tested for normality of distribution and homogeneity of variance before analyzing. Statistical difference between two groups were assessed by Student’s *t*-test. Data that met normality and homoscedasticity were evaluated with one-way ANOVA followed by Student-Newman-Keuls (S-N-K) comparison among multiple groups. Otherwise, data were analyzed with non-parametric tests. *p* < 0.05 was considered statistically significant.
